# From Lab to Zoom: Adapting Training Study Methodologies to Remote Conditions

**DOI:** 10.3389/fpsyg.2021.694728

**Published:** 2021-07-19

**Authors:** Valerie P. Bambha, Marianella Casasola

**Affiliations:** Play and Learning Lab, Department of Psychology, Cornell University, Ithaca, NY, United States

**Keywords:** Zoom, preschool, training study, remote testing, learning, spatial skills, visuo-motor integration

## Abstract

Training studies extend developmental research beyond single-session lab tasks by evaluating how particular experiences influence developmental changes over time. This methodology is highly interactive and typically requires experimenters to have easy, in-person access to large groups of children. When constraints were placed on in-person data collection due to the COVID-19 pandemic, administering this study format in the conventional manner became unfeasible. To implement this type of research under these new circumstances, we devised an alternative approach that enabled us to conduct a live, multi-session training study using a diverse array of activities through an online interface, a task necessitating creative problem solving, since most existing remote methodologies either rely on unsupervised methods or have been limited to single sessions and restricted to a limited number of tasks. The current paper describes the technological and practical adaptations implemented in our online training study of 118 4- and 5-year-old children from a geographically diverse sample. An experimenter interacted with the children once a week for 5 weeks over Zoom. The first and final sessions were dedicated to collecting baseline and post-test measures, while the intermediate 3 weeks were structured as a training designed to teach children specific spatial-cognitive and visuo-motor integration skills. The assessments and training contained image-filled spatial tasks that experimenters shared on their screen, a series of hands-on activities that children completed on their own device and on paper while following experimenters’ on-screen demonstrations, and tasks requiring verbal indicators from the parent about their child’s response. The remote nature of the study presented a unique set of benefits and limitations that has the potential to inform future virtual child research, as our study used remote behavioral methods to test spatial and visuo-motor integration skills that have typically only been assessed in lab settings. Results are discussed in relation to in-lab studies to establish the viability of testing these skills virtually. As our design entailed continual management of communication issues among researchers, parents, and child participants, strategies for streamlined researcher training, diverse online recruitment, and stimuli creation are also discussed.

## Introduction

The integral role of experience is studied in lab settings through *training studies*, a multi-session methodology in which experimenters first assess children at baseline on measures of interest and then, in subsequent lab sessions manipulate the types of input and experiences the children receive based on their assigned condition. At the end of the study, children are tested again on the same measures as at baseline and the results are analyzed to determine differential patterns of success across the training conditions. Training studies have been effectively applied to a wide variety of developmental domains, such as social cognition, language development, mathematical cognition, spatial skills, working memory, and the development of positive psychological traits such as optimism (Hale and Tager-Flusberg, 2003; Uttal et al., 2013; Hofmann et al., 2016; Gade et al., 2017; Malouff and Schutte, 2017; Casasola et al., 2020; Mix et al., 2020). Their study design makes it possible to draw causal conclusions about what specific aspects of these controlled experiences lead to improvements in particular kinds of skills.

Training studies typically require that experimenters have access to large groups of children in person over an extended period of time. However, even in ordinary circumstances this type of recruitment is challenging, as researchers must pull and retain a sufficient sample from the limited geographic areas surrounding their universities. Following the beginning of the March 2020 social distancing restrictions in the United States due to the COVID-19 pandemic, in-person data collection at most universities was stopped completely or severely restricted. As of May 2021, in-person data collection remains limited at most institutions across the country, especially with vulnerable populations such as children. It therefore became necessary for child researchers to think of creative solutions to translate their methods to online platforms. This problem was particularly messy for experimenters who wanted to maintain the hands-on and longitudinal nature of training studies.

While remote methodologies for developmental research exist that predate the COVID-19 era, they are not the most suitable for training studies that seek to provide children with controlled, multimodal, and interactive experiences that target a range of skills over multiple sessions. Most established remote methodologies were intended to be implemented in the absence of a live experimenter (Scott and Schulz, 2017; Rhodes et al., 2020). For example, parents and children using the online Lookit platform^1^ from Scott and Schulz (2017) never interact directly with an experimenter. Instead, infants and children watch videos of prerecorded stimuli, and their eye gaze is captured and saved through their webcam. Likewise, studies implemented through Discoveries Online,^2^ an unmoderated interface designed for verbal children ages three and older, participants make selections on their screen based on study narratives and animations (Rhodes et al., 2020). Although these methods allow families to complete sessions at their convenience, they do not lend themselves well to tasks requiring the child’s active participation, as during Lookit tasks the child simply watches the screen and is not able to interact directly with anything they see, and children participating in studies from Discoveries Online are constrained to actions that can be elicited with little setup and explanation, such as pressing a button on the screen or discussing a story with their parent in a naturalistic setting (Scott and Schulz, 2017; Rhodes et al., 2020). Because there is no live experimenter in either methodology, there is no mechanism in place to ensure that the instructions are followed, the child remains engaged and fixated on the screen, the camera angles stay in focus, and the data upload correctly. This last point is particularly pertinent because without an experimenter present to assume the responsibility of recording and administering the session, approximately 35% of the Lookit videos analyzed in Scott and Schulz (2017) were unusable, the majority due to missing or incomplete video data. Additionally, although Rhodes et al. (2020) reported a low level of parental interference in the studies conducted on their Discoveries Online platform, it is important to note that their studies that were not explicitly about parent-child interactions and were intentionally designed to require as little parental involvement as possible. Though such a setup reduces the risk of parental interference, it is not well suited for the goals of a training study that require child engagement in specific activities over several sessions.

Moreover, one of the only existing empirical studies that has used live videoconferencing to interface with children, Roseberry et al. (2014), a language learning study that examined whether social contingency would aid toddlers’ ability to learn words from digital applications such as Skype, was conducted in a single session in a lab setting that only used videoconferencing for a small portion of the session, and only for children in one of the three study conditions. This video chat was supplemented by a warm-up period during which the child was able to play with toys and meet the experimenters face to face, and in-person data collection methods such as eye-tracking using a physical eye-tracker. The videoconferencing component itself was also not entirely interactive, as children participated in short verbal exchanges with the experimenter at the beginning of the chat, but transitioned to passively watching and listening to the experimenter during the actual word-learning tasks (Roseberry et al., 2014). The children did not complete any participatory activities related to the word-learning task or engage the experimenter in conversation about the novel words. Established remote methodologies for developmental research have therefore mostly been applied to tasks in a narrow range of domains and modalities that are meant to capture either implicit measures or the impact of limited forms of interaction that are not directly related to the skill the child is learning.

By contrast, hands-on training studies that teach children specific skills through distinct, multimodal activities over multiple sessions have not yet been attempted in remote settings. To successfully carry out such a study, researchers would have to create study stimuli and activities that allow children to actively participate in a virtual environment, find enough families that are willing to commit to several live, online study appointments, and maintain efficient and effective communication between families and the research team, all while fostering an interactive and engaging atmosphere during the sessions themselves. The present paper details the novel approach that our research team adopted to address these obstacles in a spatial training study with 118 preschool children. In addition to being the first instance of an entirely remote training study, our study was the first of its kind to test spatial-cognitive and visuomotor integration skills, which generally rely heavily on physical materials or detailed eye-tracking methods, through behavioral methods administered through virtual interactions with experimenters. It featured baseline and post-test assessments on a variety of spatial and visuo-motor integration skills, as well as trainings with hands-on drawing activities. We will review our study’s strategy for (1) participant recruitment, (2) stimuli creation and piloting, (3) study procedure and task structure, (4) long-distance research team training, and (5) parent communication. Although this approach was devised out of necessity, we believe its takeaways can be applied to future developmental studies to overcome some of the traditional recruitment limitations in the field, such as lack of geographical diversity, and expand the reach of our science. However, it is also important to note that even though our team was successful in applying remote methods and obtaining quality data, we cannot assume that the research experiences children received over Zoom is comparable to the usual in-person experience. Future work is needed to more directly compare the patterns and quality of data obtained in remote and in-person developmental studies.

## Participant Recruitment

Our final sample size was 118 participants (65 girls, *M* = 5.05, *SD* = 0.517, range = 3.78–5.94 years). The study was conducted in two five-week rounds with different participants. Just over half of this sample participated in the five-week study in the summer (*N* = 67, 36 girls, *M* = 5.0, *SD* = 0.510, range = 3.78–5.90 years) while the remaining participants completed the five-week study in the fall (*N* = 51, 29 girls, *M* = 5.10, *SD* = 0.51, range = 4.16–5.94 years). Most participants (*N* = 105) were recruited from public and private Facebook groups designed for parents looking for virtual activities during the pandemic or online homeschooling resources for their children. All Facebook recruitment was handled by the first author. When asking permission to join a private Facebook group we made our intentions to advertise the study clear in our request form. Our ad contained an image and text describing the purpose, age requirements, format, length, and compensation for the study.

Interested parents replied to the lab email address, commented on the post, or messaged the first author directly. If a parent left a comment indicating that they were interested in having their child participate but did not email the lab or send a private Facebook message, the first author began communication by sending the parent a message first. After the initial contact the first author sent a follow-up email or message with more detailed information about the study, including the materials needed (two separate electronic screens were required, with a preference that one be a tablet), an explanation of the links they would be receiving from their experimenter containing the activities, a reminder of the study format, length, and compensation, and the projected start date of the study with a request that parents send three ranked day and time preferences (in Eastern Time) for their sessions. For organizational purposes, we asked the parents to try to pick time slots at the same time and on the same day of the week each week for each of the 5 weeks. After a timeslot had been decided, the first author then connected the family with the member of the research team who would be running their sessions. Any future communication about rescheduling was coordinated by that researcher.

Our sample was geographically diverse, with 8 participants from the New England Region, 21 from the Mid-Atlantic Region, 16 from the greater Washington Metropolitan Area where the first author is from (DC, Maryland, Delaware, West Virginia, and Virginia), 10 from the Southeastern Region (North Carolina, South Carolina, Georgia, and Florida), 8 from the Southwestern Region (Arizona, Texas, Oklahoma, Arkansas, and Louisiana), 28 from the Midwest, 10 from the Rocky Mountain Region, and 15 from the Pacific Region. Two participants did not report location information.

Of the children whose caregivers reported racial demographic information, 87 were Caucasian, 14 were mixed race, 1 was American Native or Alaska Native, 1 was African American, 11 were Asian, and 1 was Native Hawaiian or other Pacific Islander. Fifteen of these children were reported as Hispanic or Latino. Of the 118 families who reported maternal education, all had graduated high school and 112 had earned at least a 4-year degree.

An additional 31 children were recruited but were not included in the final sample due to failure to begin the study after setting up a timeslot (*n* = 10), failure to complete all five sessions of the study after starting (*n* = 4), parental involvement (*n* = 11), technological difficulties (*n* = 1), or fussiness (*n* = 5).

We were able to recruit a larger sample with less attrition in the summer (*n* = 80 recruited, *n* = 67 participated) compared to the fall (*n* = 72 recruited, *n* = 51 participated), possibly because families had more free time during the summer to dedicate to our study rather than during the fall when children had the added commitment of school.

Written informed consent was obtained from a parent or guardian before the first session of the study. All procedures involving human subjects were approved by the Institutional Review Board.

Families were given a total of $25 in electronic Amazon gift cards, $5 after their first session and $20 after their final session. In order to receive the full $25 families had to complete all five sessions.

## Stimuli and Procedure

### Baseline and Post-test Assessments

The baseline and post-test contained a total of six assessments on a variety of spatial, language, and visuo-motor integration skills. Three of these measures were administered through Qualtrics, two were administered through another online behavioral science platform called Gorilla,^3^ and one was administered through virtual demonstration and a physical writing instrument and paper. We used two online platforms to more closely approximate versions of tasks that had been conducted successfully in person (Qualtrics tasks) and to administer the same version of tasks that are being used in a different ongoing online study in our lab with the comparatively older age group that was the focus of this study, setting the stage for future age comparisons (Gorilla tasks). The remaining task was adapted from a standardized visuo-motor integration task, so we used physical materials to more closely mimic its standard setup.

**Figure 1:**
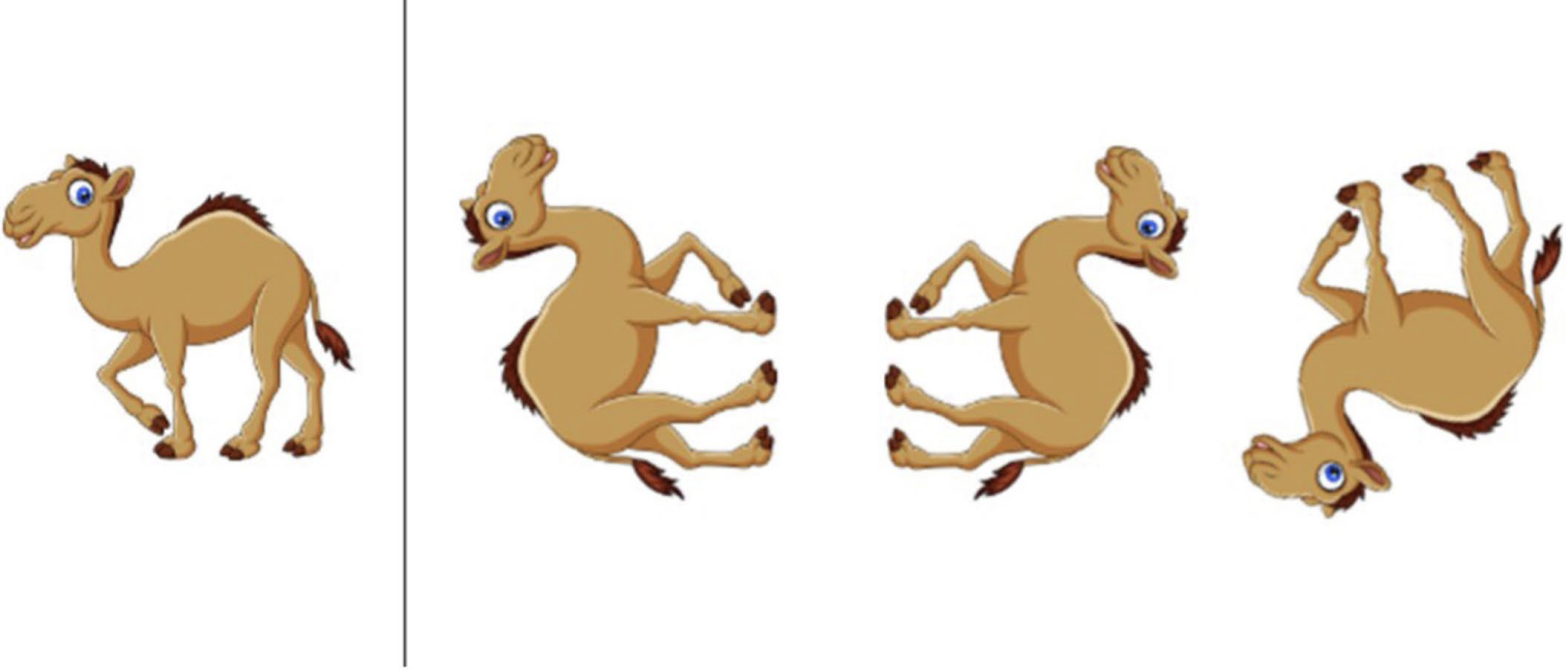
Example trial from the mental rotation task.

The first assessment was a mental rotation task based on the picture rotation task (PRT) used in Quaiser-Pohl (2003). There were two versions of the task, modeled on the two versions of the standard PRT but using different images. Children received one of the two versions at baseline and the other at post-test. During the task, they had to identify which of three rotated images exactly matched an example image. This task was administered as a Qualtrics survey that an experimenter displayed through screen share on Zoom (see Figure 1). Across both rounds of the study, children answered by pointing to a numbered picture and having their parent tell the experimenter which picture they chose (*n* = 46), by verbally responding themselves (*n* = 71), or by listening as the experimenter labeled each of the choices and telling them to stop when they came to the picture that they thought was the correct match (*n* = 1). The task contained three practice trials in which the child was always shown the correct answer and 12 test trials where the correct answer was not shown.

**Figure 2:**
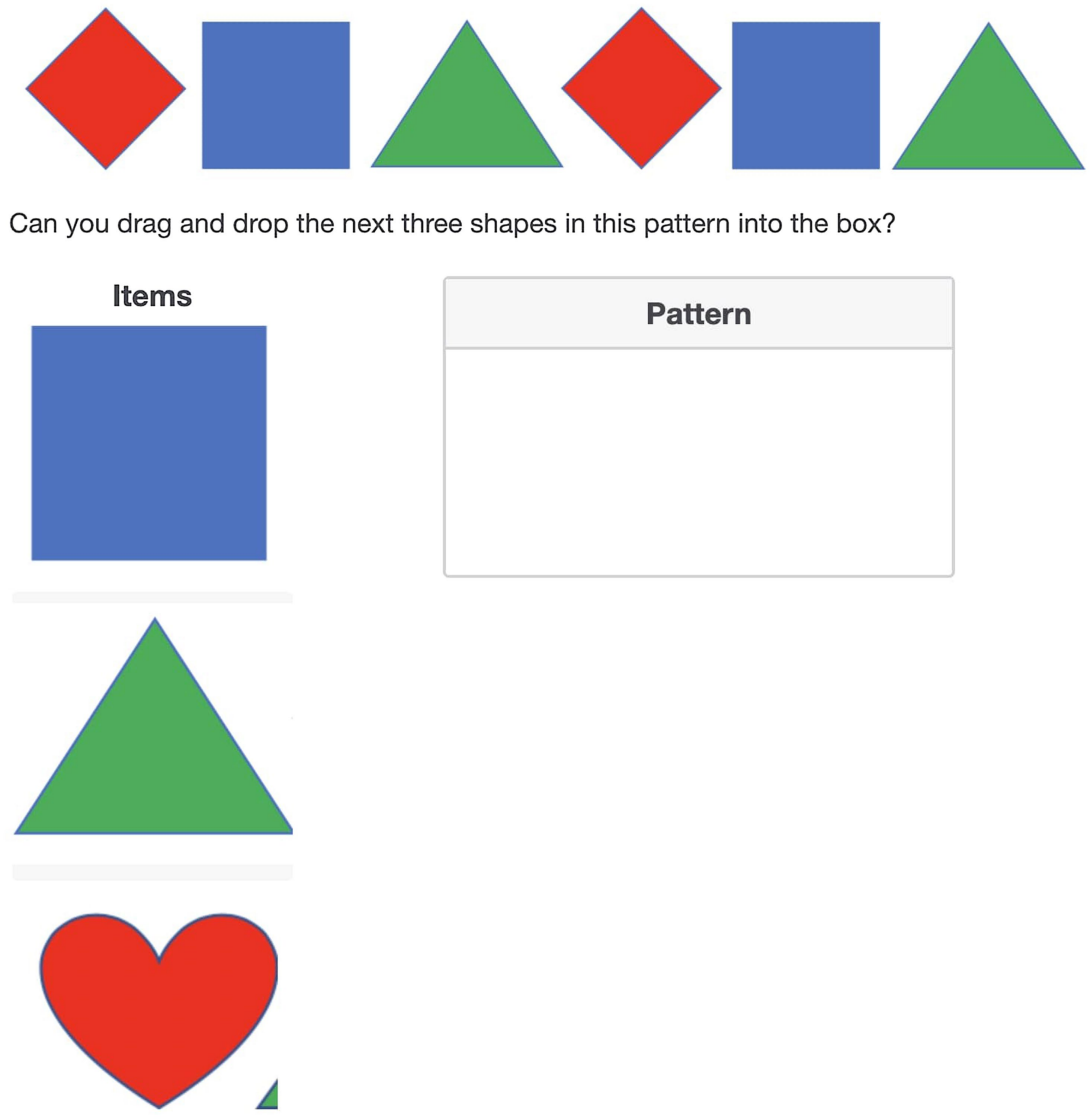
Example trial from the pattern task.

The second assessment was a novel pattern extension task that was also administered through a Qualtrics survey (see Figure 2). There were two versions of this task with patterns with similar structures but different specific images. Children completed one version of this task at baseline and the other at post-test. This task was conducted on a second device while the experimenter followed along by displaying the corresponding screens through screen share on Zoom. Parents were sent the survey link prior to the testing session and had the task ready for the child to complete with the experimenter during the session. The task required children to both verbally indicate and drag and drop the three elements that came next in a series of six patterns into an answer box. They completed the task on their second device. Experimenters did not select any answers for the children during this task. Instead, children whose second device had a touchscreen (*n* = 100) used their finger to move the pictures into their correct positions in the pattern, with parental assistance as needed. Children whose second device was a laptop with no touchscreen (*n* = 18) indicated their answers by pointing to the picture on the screen and having their parent complete the drag and drop for them.

**Figure 3:**
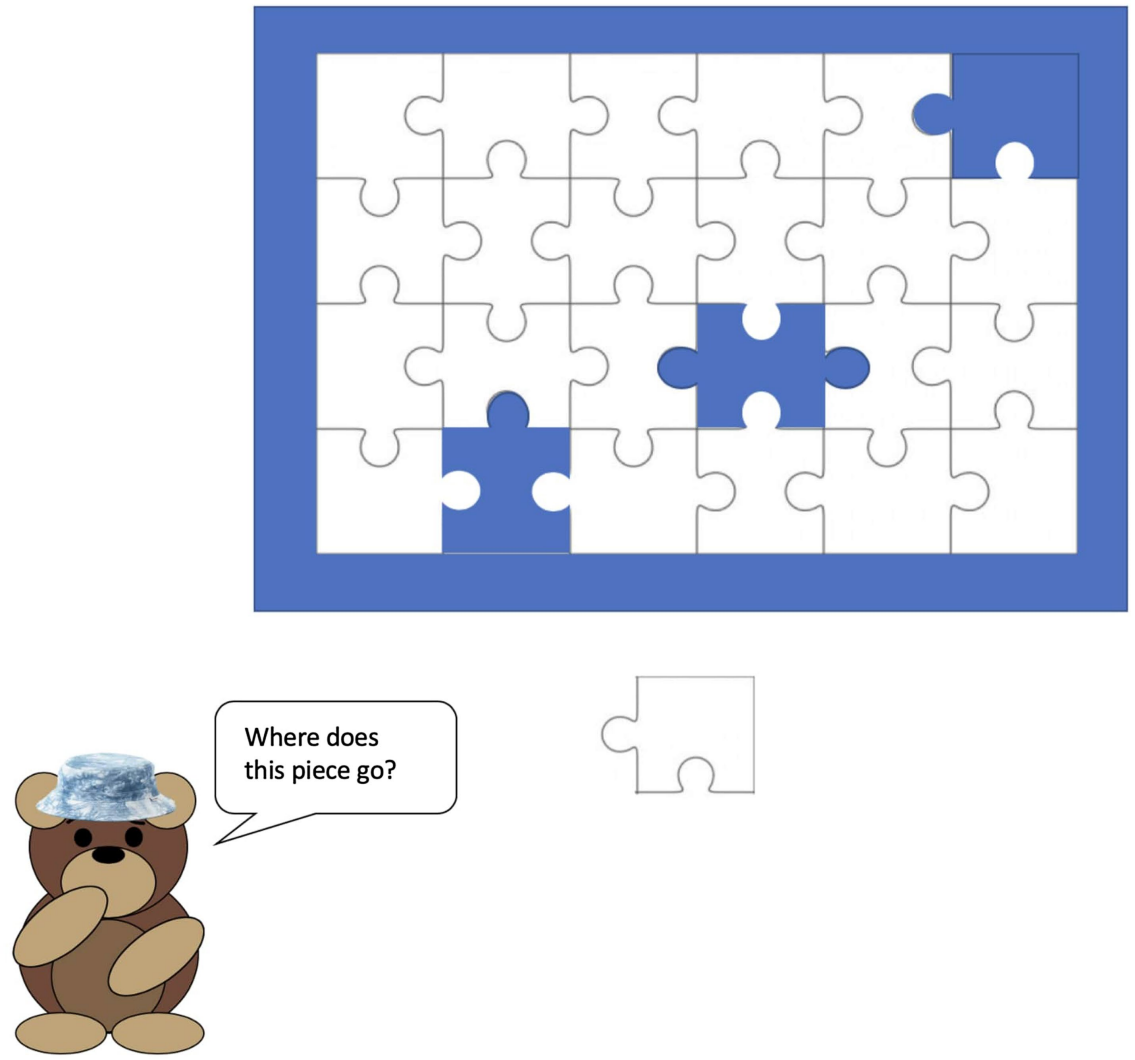
Example trial from the puzzle task.

The third assessment also took place on the child’s second device but was instead administered through the Gorilla platform. Children were shown a series of nine partially completed puzzles and were told to tap on the space where they thought a missing piece went (see Figure 3). There was no drag and drop element involved. Children whose second device had a touchscreen (*n* = 100) used their finger to tap on the matching space while those whose second device did not have a touchscreen (*n* = 18) pointed to the spot they thought was correct and their parent clicked it for them.

**Figure 4:**
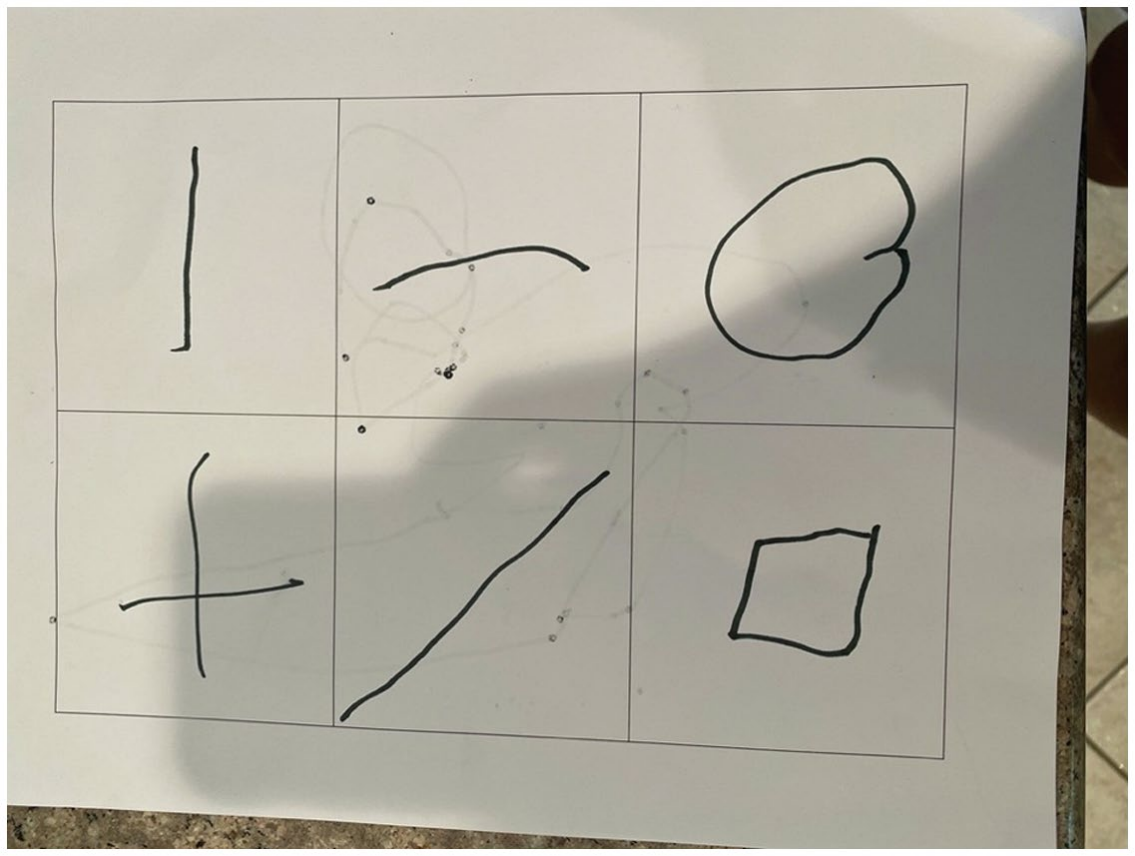
Example images from the modified Beery DTVMI task.

Children completed the fourth assessment using a physical drawing tool and paper. For this assessment, which was a modified version of the Beery Developmental Test of Visuo-Motor Integration (DTVMI) from Beery (2004), the experimenter held up a series of geometric images to the screen and had the child copy them into sheets of paper that contained tables with two rows and three columns (see Figure 4). The child held each page up to the screen once they had filled the table. This table had been emailed to parents the night before. There was a total of 15 progressively more difficult images for the child to copy, but the experimenter stopped early if the child was unable to draw an image or expressed a desire to stop. Children completed on average 12 drawings at baseline and 13 drawings at post-test.

**Figure 5:**
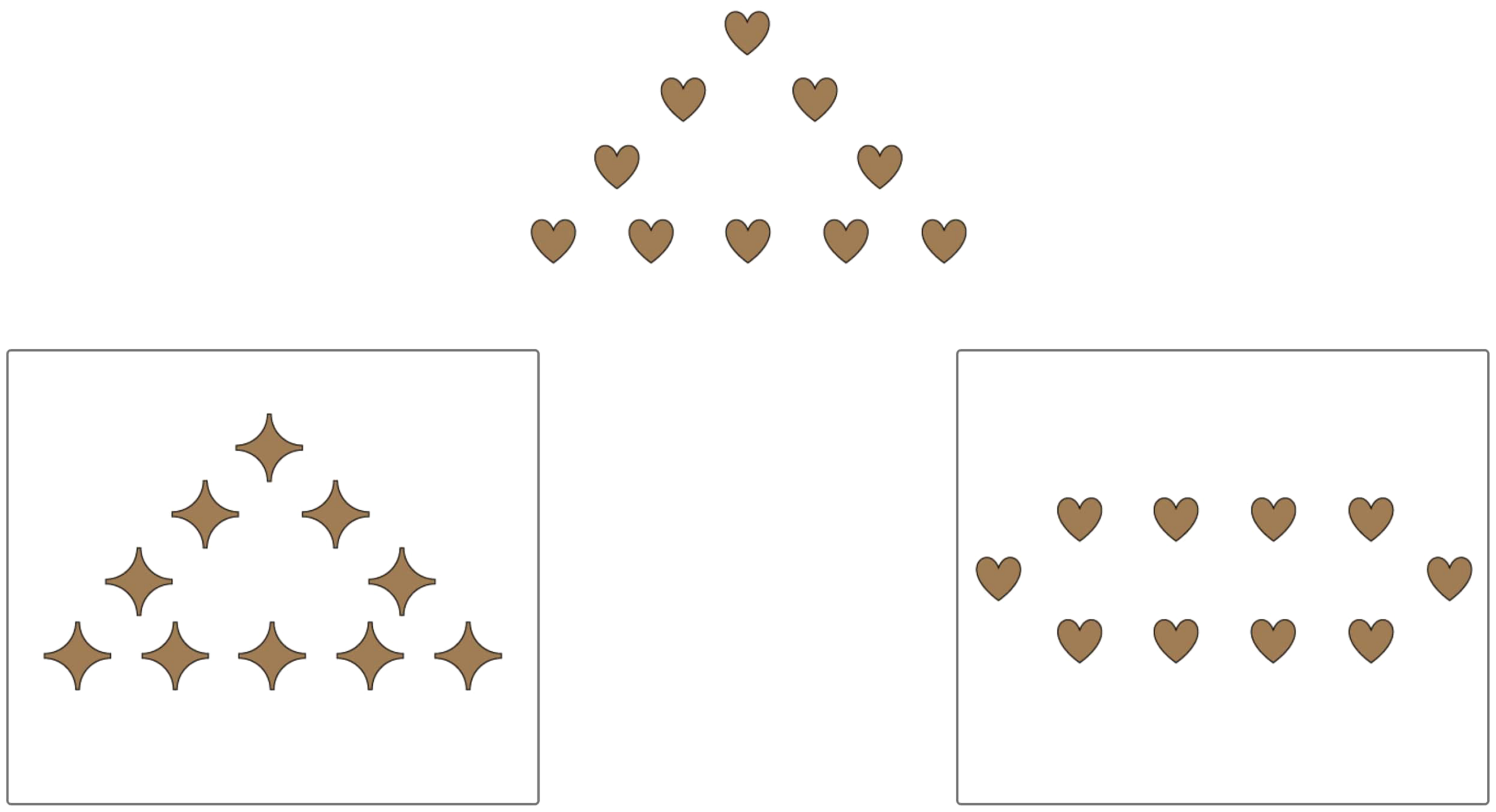
Example trial from visual processing task.

For the fifth assessment, children returned to Gorilla on their second device. This task was a test of visual processing and required them to pick which of two pictures at the bottom of the screen they thought looked the most like the picture at the top of the screen (see Figure 5). Children whose second screen was a touchscreen (*n* = 100) used their finger to select their choice while those whose second screen was a laptop with no touchscreen (*n* = 18) pointed to their choice while their parent clicked it.

**Figure 6:**
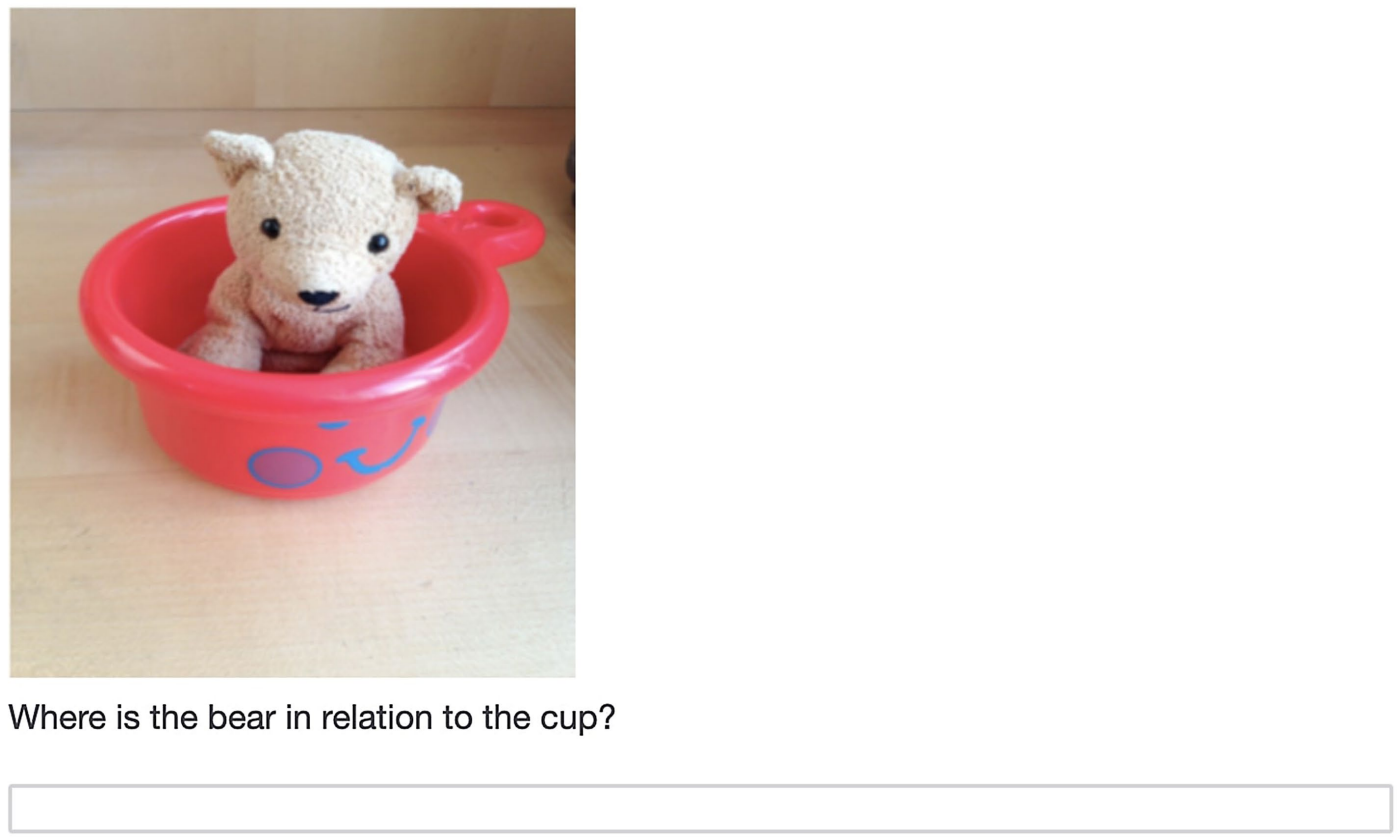
Example trial from the expressive spatial relation portion of the spatial vocabulary assessment.

The sixth assessment was a spatial vocabulary task administered in a Qualtrics survey over screen share. There was only one version of this task and it contained items that ranged in difficulty. For the expressive part of the task, an experimenter shared pictures with various geometric shapes and spatial relations and asked the child to verbally label them (see Figure 6). The experimenter recorded the child’s response directly into the form (24 total questions: 15 shape and 9 spatial relation). During the receptive part of the task, the experimenter provided the label and children had to select the corresponding picture (21 total questions: 9 shape and 12 spatial relation). For this part, 76 children responded by pointing to one of the numbered choices on the screen and having their parent tells the experimenter which one they pointed to and for 29 children the experimenter verbally scanned through the numbered options and told the child to tell them to stop when they landed on the correct choice. Twelve children responded on their own.

All children were asked to complete a free draw after they completed the sixth assessment. Depending on children’s level of engagement, the experimenter sometimes presented the tasks out of order or gave the child a break to complete an extra free draw. For example, if a child started to lose focus during the interactive drag and drop task the experimenter would either move on to a less demanding task or let the child draw a picture until they regained focus and enthusiasm. There were 36 participants who were given an altered task order in this manner.

Parents were asked to take pictures of and email copies of all physical drawings their children created to the research team. Of the 118 total participants, 71 emailed all the necessary materials for both baseline and post-test. For children without emailed materials, coding was done based on the recorded Zoom video.

### Training Activities

**Figure 7:**
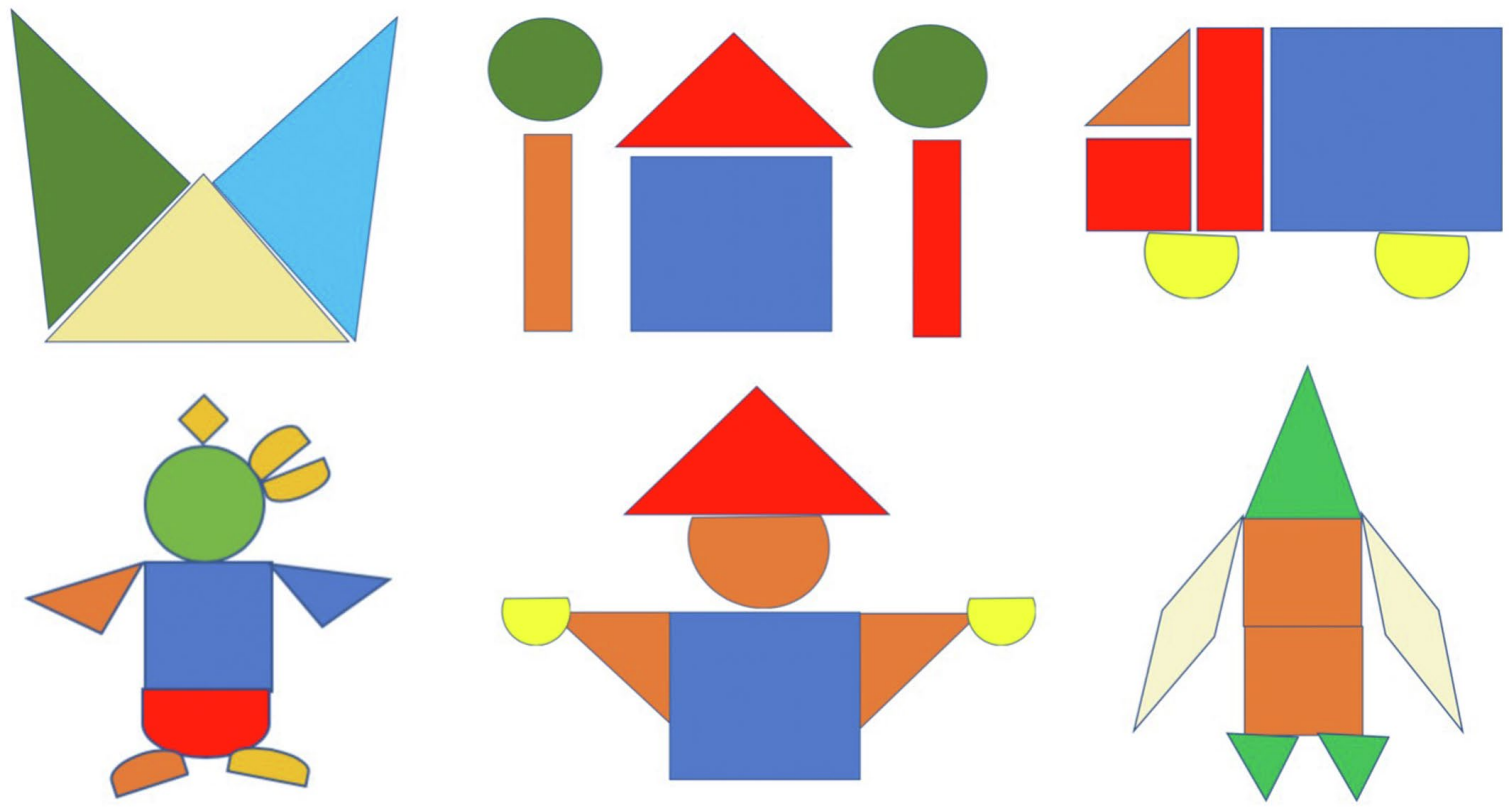
Images completed by children during their training sessions. All children received the cat and penguin images for their first training, the house and person images for their second training, and the truck and rocket images for their third training.

The training sessions were novel tasks administered through screen share by an experimenter using the Gorilla platform. Gorilla was chosen for the demonstration because it contained a drawing tool that allowed the experimenter to draw on the screen. Children were shown and asked to trace or copy two images containing geometric shapes during the guided drawing portion of these training sessions. All children were given the same images in the same order for each session. The images for the first training session were the cat face and the penguin, the images for the second were the house with trees and the person, and the images for the third training session were the truck and the rocket (see Figure 7). Some children were provided with informative spatial language while they completed the art activities, and some were not.

After the two guided draws, all children completed two free draws. For the first free draw, they were instructed to draw whatever they wanted. For the second, they were told to draw whatever they wanted using as many different kinds of shapes as they could.

Parents were asked to take pictures of and email copies of all physical drawings their children created to the research team. Of the 118 total participants, 73 emailed all the necessary materials for all three trainings. For children without emailed materials, coding was done based on the recorded Zoom video.

## Research Assistant Training

A total of 12 undergraduate research assistants, along with a hired lab manager and the graduate student principal investigator, interacted directly with the children over Zoom during data collection and assisted with behavioral coding and data processing. Three additional undergraduate research assistants worked solely on behavioral coding and data processing.

In order to maintain uniformity with such a large team that could not gather in person and that had members located in different time zones due to the unique situation created by COVID-19, we set up a series of Zoom trainings and created detailed step-by-step guides stored in our lab Box folder that contained instructions for proper data collection protocol and links to needed materials. Research assistants also clearly marked their availability in a shared Google calendar. This organizational process proved crucial in ensuring that all the research assistants were well trained and able to carry out the protocol smoothly.

Data collection took place in two phases: summer and fall. During summer session, the first author graduate student and five undergraduate research assistants conducted sessions with 67 children, with a range of 6 to 17 participants per researcher and a total of five sessions per participant. The undergraduate students met with the graduate student over Zoom for an initial training where they were walked through the procedure over screen share and shown where the guides and materials were stored. The first author began running participants a week before the undergraduate students, and as part of their training, the undergraduates were required to shadow the first author at least once while she ran her baseline sessions by joining as a participant on the Zoom call. As the first author remained a week ahead of the undergraduates, they were instructed to continue shadowing at least once a week in preparation for the next week’s procedure and to review the first author’s videoed sessions, which were also stored in the lab Box folder. The first author also remained in contact with the undergraduates *via* email, set up personal Zoom meetings to answer any questions they had about the procedure, and shadowed sessions upon request. The first author or another research assistant served as substitutes if a researcher had to miss a session for any reason.

Six new undergraduate research assistants joined the research team in the fall, and one experienced undergraduate left. Fall data collection was conducted by these undergraduate research assistants, the graduate student first author, and the lab manager for a total of 51 participants with a range of 1 to 9 participants per researcher and a total of five sessions per participant. As in the summer, the new undergraduate research assistants met with the first author over Zoom for an initial training. New research assistants were also paired with an experienced research assistant who had collected data over the summer. The purpose of these pairings was to provide new research assistants with an accessible resource who could help answer questions and troubleshoot difficulties faster than if they had to rely solely on the first author. Fall data collection began at the same time for all researchers, but the new research assistants had to shadow the experienced research assistant they were paired with at least once a week. The experienced research assistants were also expected to shadow their new research assistant at least once a week to ensure they were conducting their session correctly. The first author remained available for questions that could not be answered by the more experienced research assistant and shadowed sessions upon request. The lab manager and the first author served as substitutes if a researcher had to miss a session for any reason.

Coding guides were created and placed in the lab Box folder detailing the necessary steps for data coding and processing for each of the tasks. Research assistants involved in data collection were expected to fulfill the remaining hours they had committed to the lab by working on coding. Three undergraduate research assistants worked only on coding and data processing. The first author set up individual Zoom meetings with each of the coders as questions arose and clarified instructions further over email.

Six undergraduate research assistants who completed an open-ended survey about their experience with remote data collection over Zoom named scheduling flexibility and geographic diversity as advantages of the approach and also said that the children seemed to be engaged with the tasks overall. The fact that the child was in their own home with their family was described as both an advantage since the child was more comfortable and did not need to warm-up as much to the experimenter, and a disadvantage since it was more difficult for the experimenter to establish authority and redirect children’s attention from behind a computer screen. Respondents also said that relying on parents to redirect the child, access the needed links, and adjust camera angles was challenging, although it was easier to coordinate rescheduling sessions than when we run in-person studies in the lab. They also wrote that technical difficulties arose occasionally for both experimenters and families, but rarely significantly impacted the sessions.

## Communication with Parents

Our team found that consistent communication with parents was key for participant retention in this multi-session study. We emailed parents the night before each of their sessions, which served both to remind them about their upcoming session and make them aware of what materials they would need, where they were located, and what they would need to do to prepare.

### Communication for Baseline and Post-test Assessments

Parents were sent an email the night before their scheduled session with the Gorilla.sc link they would need to access the consent form, demographic survey, and tasks. They were also sent an additional Qualtrics link that contained the pattern task. This email also included a file with the table that the children would need for the activity requiring paper and a physical drawing tool, instructions about how to fill in their child’s participant ID and other information in the forms they were sent, which device they should access each form on, and a description of the physical materials they would need for the session. Parents were also reminded that they needed to be present during the session and that they should offer encouragement, but no hints, to their children and that they would need to take pictures of anything their child physically drew and email them to the lab. Lastly, the email mentioned that they would receive a Zoom link 10–15 minutes before the scheduled start of their session, where their researcher would be watching and recording from.

### Communication for Training Activities

Parents also received an email the night before each of their scheduled training sessions. Parents whose children were in the condition where they would be asked to trace images received a file with those images in this email and were instructed to copy them in pencil on two separate sheets of paper. They were told not to show these images to their children until the start of the session. All parents were told what materials they needed, were reminded to expect a Zoom link 10–15 minutes before the scheduled start of their session, and were told to take pictures of all their child’s drawings and email them to the lab.

## Advantages and Disadvantages of Remote Data Collection For Training Studies

The COVID-19 pandemic necessitated substantial adaptations to established training study methodologies. For our specific study, our main challenge was maintaining a controlled and standardized procedure across children’s diverse home environments, which we could not physically manipulate, and across multiple sessions. The remote format of the study meant that the stimuli and setup of the study space, usually the responsibility of the researcher, now fell on the parent. While we tried to provide parents with detailed instructions, most parents do not have formal research background and are often trying to set up the study in a hurry. Additionally, because families’ participation is a significant service to us, we did not want to overburden them with instructions that were too cumbersome or difficult to understand. Striking the appropriate balance required trial and error, and though we were able to maintain a higher level of standardization through our interactions with parents than unsupervised remote methodologies, there were still some elements that were ultimately out of our control despite providing instructions, such as what a parent chose to say to their child during the session (*n* = 2 participants received some form of direct parental prompting about which shapes to draw during their free draw; *n* = 11 participants were excluded from analysis for at least one baseline or post-test assessment due to excessive prompting), whether the parent was present during the session at all (*n* = 22 participants had no parents present for at least one of their training sessions), technological difficulties (*n* = 1 participant was excluded from analysis of at least one task due to technological difficulties), and the child’s attention (*n* = 5 participants were excluded due to fussiness). We did find that giving explicit instructions to parents about how to engage during the sessions, both in writing before the sessions and verbally during the sessions, was helpful in ensuring that the study protocol was followed. It is also worthwhile to note that although our study relied heavily on technology only one participant was excluded due to technological difficulties. Technology issues, such as slow Internet on either the experimenter’s or participant’s side, occasionally arose but were able to be resolved by using a Wi-Fi hotspot, restarting Zoom, or rescheduling if necessary. Internet problems were therefore not a significant impediment to data collection. However, as families were aware of the technological requirements before starting the study, it is likely that we mostly attracted families who believed they would have stable Internet.

The members of our research team were also trained extensively on what to do when they encountered issues, such as on how to verbally label answer options for the child if their parent was not present to indicate which option they had pointed to, how to provide guidance to parents about how to adjust their camera angle, suggestions for helping the parent interact with their child in a way that aligned with study protocol, solutions to common technological difficulties, and strategies for redirecting children’s attention when they started to lose focus. These adaptations were necessary to maximize usable data and enabled us to offset procedural issues that arose from uncontrolled environments and that would have led to some participants being excluded from analysis of certain tasks. However, we did exclude more participants in this study compared to comparable in-person training studies carried out by our lab: *n* = 31 participants excluded in this study out of a total of 149 participants recruited compared to *n* = 11 participants excluded out of a total of 95 recruited participants in Casasola et al. (2020).

Overall, our team found that the study tasks were engaging for children and worked well virtually, though there were a few notable challenges and general observations. We observed that children were more able to independently complete certain types of tasks than others. For example, the majority of children was able to complete the Gorilla touchscreen tasks that required a single tap on the correct answer on their own, but many seemed to struggle completing touchscreen tasks requiring more exact motor control without parental assistance. Children often became frustrated when they were not able to complete a task on their own. Children also appeared to be the most focused when they were completing a task that involved a level of participation and motor engagement from them that was neither too little nor too taxing. For example, children seemed on task when they were drawing or tracing during the training sessions and the modified Beery DTVMI assessment, selecting the matching image from the three options in the mental rotation task, or answering questions about spatial vocabulary, but at times appeared to speed through the single-tap Gorilla tasks and became frustrated by pattern extension task, which required considerable manipulation of the touchscreen. These differences suggest that children engage well both when the tasks are administered by an experimenter through screen share and when they are able to manipulate physical drawing materials but can struggle maintaining focus when asked to interact directly with a touchscreen. These observations are anecdotal and should be explored further in relation to age differences and individual differences in attention, fine motor skill, and technology exposure. While the study activities and multi-session setup worked generally well for children in the age range we used, it is an open question as to whether younger children would be able to engage in a multi-session online study with these types of interactive activities. Results would be informative about the most effective remote training methodologies for teaching spatial-cognitive skills to children across a wide age range.

Using both Qualtrics and Gorilla to conduct our screen-based tasks allowed us to make comparisons about how well the two platforms hosted our baseline and post-test assessments and our interactive training activities. As a reminder, we used Qualtrics forms for the mental rotation, pattern extension, and spatial vocabulary tasks and Gorilla for the consent form and demographics, puzzle completion task, visual processing matching task, and the tracing and drawing demonstrations during the training sessions. The biggest difference between the two platforms was the amount of touch-based interactivity that was possible to integrate into each one. While the pre-made templates in Qualtrics are restricted to a few default setups (i.e., multiple choice and free answer questions, limited touch-based drag and drop matching activities) with limited aesthetic and functional customization, Gorilla has a zone feature that facilitates the creation of more complex activities in a user-friendly manner that does not require programming knowledge. This Gorilla feature was especially helpful during the training sessions because we were able to use a zone to create a space where experimenters could use their mouse to draw the study images on the screen alongside the child. We also were able to use these zones to easily create the puzzle completion and matching tasks, which required children to touch different parts of the screen. Gorilla also has templates for the more standard question formats that are also included in Qualtrics. However, one disadvantage of Gorilla compared to Qualtrics is that there is a small fee ($1.20) per participant, whereas Qualtrics was free for us to use through our university. Data were also sometimes hard to download from Gorilla compared to Qualtrics, as the servers sometimes became blocked up due to heavy volume.

In terms of setup, both the Qualtrics mental rotation and spatial vocabulary tasks were administered by an experimenter *via* screen share. Because we had to link the participants’ baseline and post-test assessments to each other, each child was assigned a random ID number that the experimenter filled out along with other basic information at the beginning of the Qualtrics forms. Parents did not have to fill out anything on Qualtrics for the screen share tasks; all necessary identification information was filled out by the experimenter like in a lab setting. However, since the pattern extension task took place on the child’s own touchscreen and not through screen share, the parent was responsible for entering the child’s ID number on their own. We found that sending the ID number to parents along with the pattern extension Qualtrics link the night before saved time during the session itself and reduced confusion. We also sent the Gorilla link the night before and instructed parents to complete the first two pages with the consent and demographics information, but not to proceed further. Parents did not have to enter an ID number into Gorilla because our research team was able to enter it from our end before sending out the link. However, parents were asked to manually input information into both platforms at some point, and in spite of the emailed instructions, some had difficulty navigating between both links and remembering which information belonged in which link. To ease the burden on parents, future remote developmental researchers should streamline their methods by limiting themselves to a single platform and reducing the amount of information they have to enter that is typically inputted by experimenters.

As mentioned previously, a notable advantage to remote data collection was our ability to recruit from a wide geographic area, allowing children in areas far from universities to participate in developmental research, an opportunity both we and they would not have had otherwise. We were also able to obtain a larger sample than we have been able to acquire in past in-person training studies (*N* = 118 participants took part in the current study compared to *N* = 84 participants that took part in Casasola et al. (2020)). It should be noted, however, that due to the technological requirements and recruitment methods we used, our sample was not very ethnically or socioeconomically diverse (*n* = 87 participants identified as White/Caucasian, *n* = 112 participants came from middle and upper socioeconomic classes, as defined by maternal education). We also recruited heavily from parenting-based Facebook groups, so the nature of our sample was impacted by the types of families that seek out those types of groups to join. Wider recruitment benefits the field by providing researchers access to samples that are more representative of the general population, and future online research should supplement the inherent geographic diversity of remote research by making a concerted effort to reach out to online communities with connections to families from a wider variety of ethnic and socioeconomic backgrounds. Online research has the potential to be integrated as a fruitful avenue of recruitment even after the pandemic, although it should be viewed as an addition rather than a substitute for in-person methods, as there are some samples that cannot effectively be reached by remote methods. For example, in addition to technological requirements, online research also requires a sufficiently quiet and spacious home environment that is not available to all families.

**Figure 8:**
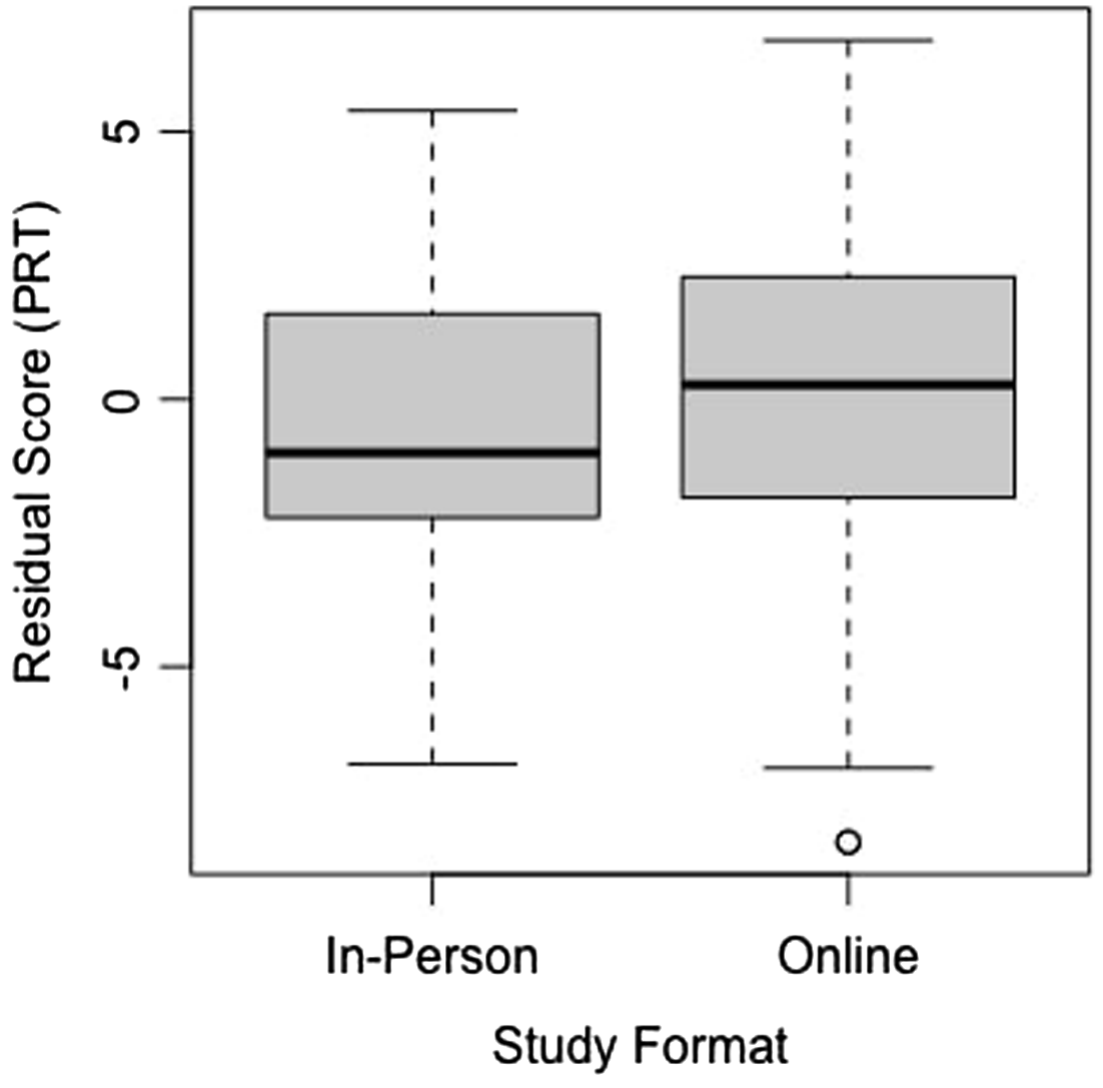
Residual scores for both formats of the mental rotation task.

Furthermore, if remote studies are to continue even after the pandemic ends, it is essential to verify that the virtual formats of tasks achieve the same internal validity as their in-person counterparts (Scott and Schulz, 2017; Rhodes et al., 2020; Oliver and Pike, 2021). Of the six baseline and post-test assessments in the current study, the mental rotation task and spatial vocabulary assessment matched in age range and format to an in-person study in our lab examining how children’s play behaviors shape their spatial skills. Both mental rotation assessments were based on the PRT from Quaiser-Pohl (2003), administered through Qualtrics, used the same number of items, and had the same scoring system. We computed the Cronbach’s alpha for both versions of the mental rotation task in the current study (Version A: *a* = 0.725; Version B: *a* = 0.713) and both versions of the mental rotation task in the in-person study (Version A: *a* = 0.768; Version B: *a* = 0.723), which indicated comparable internal reliability across the two tasks. After accounting for the effect of age by calculating residuals, two one-sided *t*-test (TOST) equivalence was calculated for the two versions of the task using the TOSTER package in R (Lakens, 2017). According to this test, we can reject effects larger than *d* = 1, *t*(86.32) = 4.893, *p* < 0.0001, suggesting that the difference between the two task formats is less than one standard deviation from zero. A boxplot depicting the overlap in the residual scores for the two versions of the task can be found in Figure 8. The statistics from the current study, which was the first to examine mental rotation through interactive online methods, produce a promising outlook on the future use of remote methodologies to test spatial-cognitive skills, as they appear to achieve equivalent effects in an online format.

**Figure 9:**
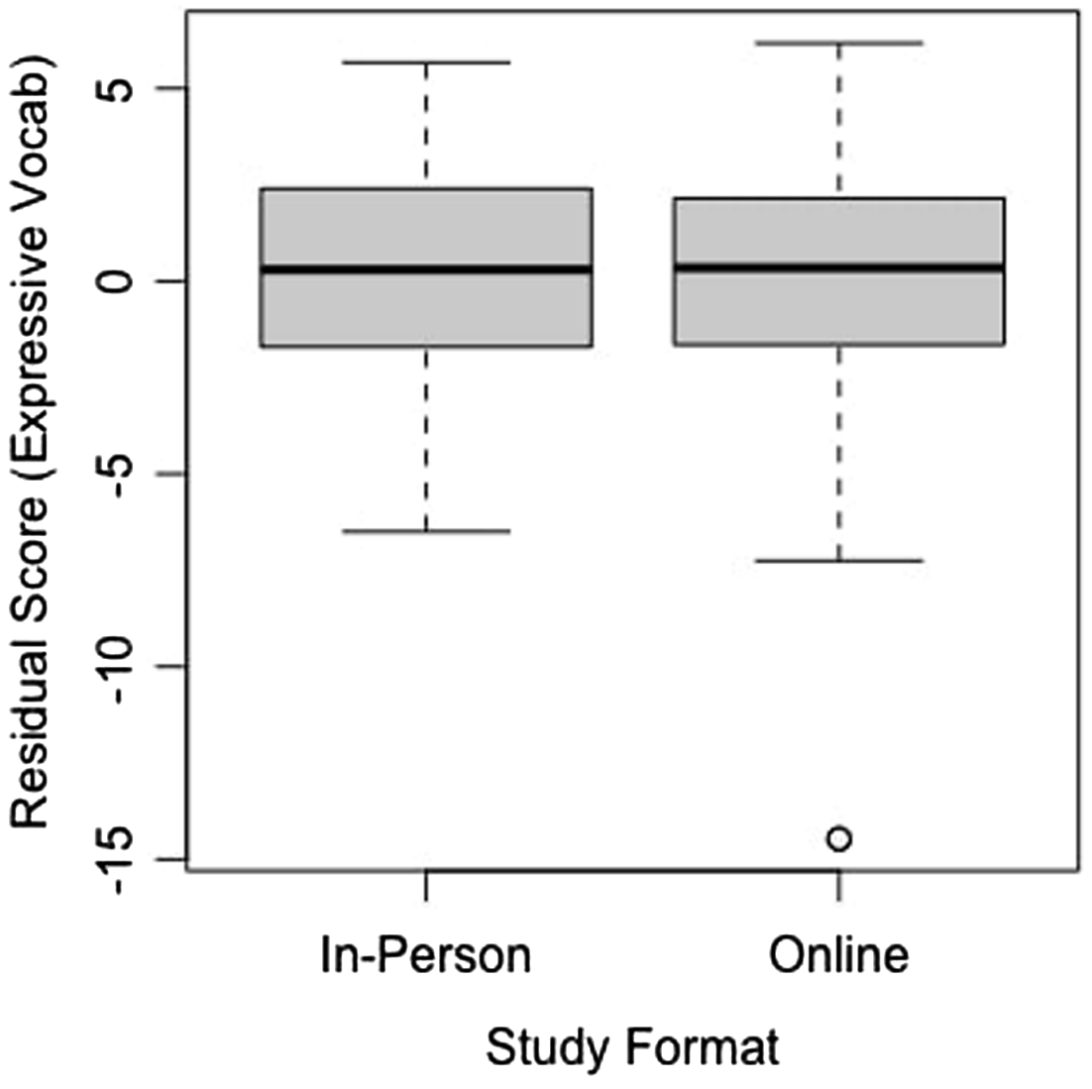
Residual scores for both formats of the expressive vocabulary section of the spatial language assessment.

**Figure 10:**
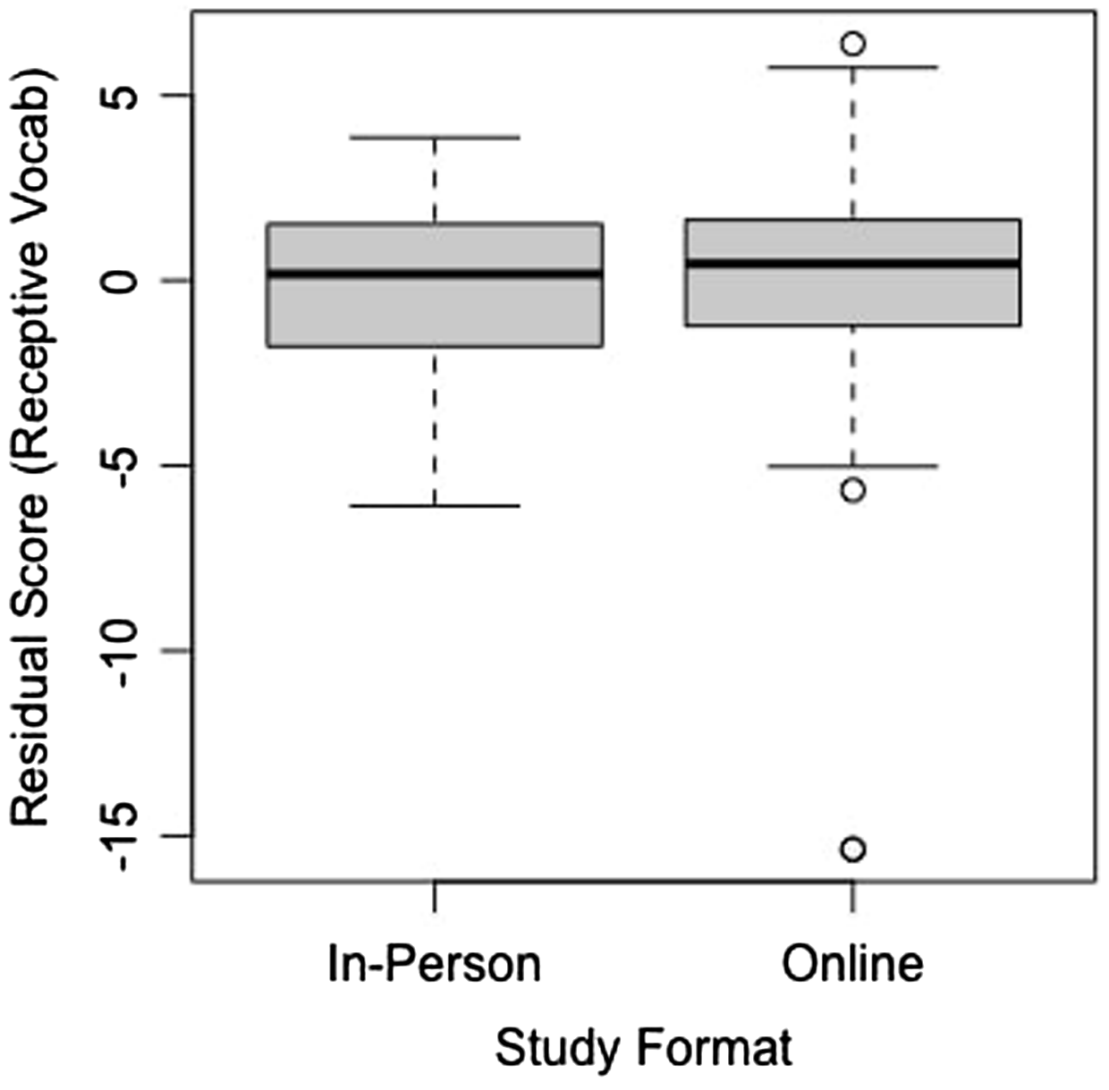
Residual scores for both formats of receptive vocabulary section of the spatial language assessment.

The spatial vocabulary assessment was created by our lab and had been administered during the same in-person study as the mental rotation task. Both assessments were on Qualtrics and contained the same items in the same order. Once again, after accounting for age by calculating residuals, TOST equivalence across the two study formats was calculated for both the expressive and receptive vocabulary portions of the assessment using TOSTER (Lakens, 2017). The results indicated that effects larger than *d* = 1, *t*(94.27) = −5.437, *p* < 0.0001 for expressive vocabulary and larger than *d* = 1, *t*(114.07) = 5.786, *p* < 0.0001 for receptive vocabulary can be rejected, suggesting that difference between task formats for both expressive and receptive vocabulary is less than one standard deviation from zero. Boxplots of the age-adjusted residuals for the two versions can be found in Figure 9 (expressive vocabulary) and Figure 10 (receptive vocabulary). It appears that children achieved similar results on the assessment regardless of whether it took place online or in-person. In line with previous remote methods that have tested language learning and knowledge virtually, this finding indicates that our spatial vocabulary assessment can be used reliably in an online format for this age group.

However, although the TOST equivalence found the two study formats to be statistically equivalent at the inputted parameters, it is noteworthy to mention that there were more extreme residual values (notably at the low end) in the online format, as can be seen from the boxplots. These values may have resulted from children becoming more distracted or less engaged in the online format and thus performing substantially below the mean for their age group. It is important to keep these possibilities in mind when interpreting data from online studies, because distractibility and engagement may not always be obvious from watching the sessions, making it difficult to successfully exclude every child who lost focus on the task.

Our remaining assessments and the training activities had no comparable in-person task from our lab for comparison (pattern extension task, Gorilla tasks, modified Beery DTVMI, and training activities). Further work is needed to compare the validity and reliability of these tasks when they are conducted in-person as opposed to a remote format, particularly for tasks, such as the modified Beery DTVMI and the drawing activities from the training studies, whose scoring involves a degree of subjectivity.

## Final Takeaways and Advice

Entering into the foray of online research, especially with a multi-session training study, introduced our team to unexpected situations that helped us develop an effective protocol for successfully conducting research remotely. As many other researchers in developmental psychology and other fields are approaching the task of adapting their own studies to this new format, we thought it would be helpful to share the more miscellaneous adaptations we employed during our sessions to ensure they proceeded according to plan. Some of this advice is specific to online studies and some would apply to either in-person or online research.

- When emailing links to parents, it is important to let them know when they can open them and how much they can fill out ahead of the session. If you do not want a parent to open a link before the session at all it is best to wait until the session begins to send it.
- Make sure parents are aware when sessions are being audio and video recorded, for what purpose, where the videos will be stored, and who will have access to them.
- It is easy for videos to get washed out, especially if the participant is sitting near a window. We always had our researchers take some time at the beginning of each session to politely ask the parent to adjust the camera until they could see what they needed to see.
- Be aware of how recording works on the platform you are using. For example, when Zoom is set to speaker view it only records video of whoever is speaking at the moment. This feature is disadvantageous when you want a video of the child and not the researcher giving instructions.
- We always had our researchers record on either gallery or spotlight view. When they had to use screen share, we had them expand the video of the participant as large as possible.
- Have your participant use darker colored crayons or markers when they have to physically draw something to ensure that you are able to see what they are drawing. Always have the child hold up whatever they are working on to the screen and make sure it is fully captured by the camera.
- Pay attention to your facial expression and offer consistent encouragement during the session. The child is most likely looking at a close-up of your face the entire time.
- It was helpful to be flexible about task order in our online format. We would recommend it if possible because it helps children maintain attention.
- Be sure to debrief the parent and child (in an age-appropriate way) at the end of the study so they know what the study was about.
- Send compensation as soon as possible after the session.
- Follow-up with parents when you know the results to give them a summary of what you found. This helps them feel included in the research process.

In short, although the widespread shift to online studies was not a voluntary one, with careful planning and study design online studies can provide a valuable source of data for developmental science that augments what researchers are able to accomplish with conventional data collection methods.

## Data Availability Statement

The raw data supporting the conclusions of this article will be made available by the authors, without undue reservation.

## Ethics Statement

The studies involving human participants were reviewed and approved by the Cornell University Institutional Review Board (IRB) protocol number: 1210003363. Written informed consent to participate in this study was provided by the participants’ legal guardian/next of kin. Written informed consent was obtained from the minor(s)’ legal guardian/next of kin for the publication of any potentially identifiable images or data included in this article.

## Author Contributions

VB and MC conceptualized the idea for the study questions and procedure. VB coordinated all details related to data collection, oversaw coding and data processing, and wrote the manuscript. MC edited the manuscript and obtained the funding used during the study. All authors contributed to the article and approved the submitted version.

### Conflict of Interest

The authors declare that the research was conducted in the absence of any commercial or financial relationships that could be construed as a potential conflict of interest.

## Footnotes

^1^www.lookit.mit.edu

^2^www.discoveriesonline.org

^3^www.gorilla.sc
